# Gingival epithelial cell-derived microvesicles activate mineralization in gingival fibroblasts

**DOI:** 10.1038/s41598-022-19732-1

**Published:** 2022-09-22

**Authors:** Shuichiro Kobayashi, Jiarui Bi, Gethin Owen, Nelli Larjava, Leeni Koivisto, Lari Häkkinen, Hannu Larjava

**Affiliations:** Faculty of Dentistry, Department of Oral Biological and Medical Sciences, University of British Columbia, Vancouver, BC V6T 1Z3 Canada

**Keywords:** Inflammation, Extracellular signalling molecules, Mechanisms of disease, Differentiation

## Abstract

Soft tissue calcification occurs in many parts of the body, including the gingival tissue. Epithelial cell-derived MVs can control many functions in fibroblasts but their role in regulating mineralization has not been explored. We hypothesized that microvesicles (MVs) derived from gingival epithelial cells could regulate calcification of gingival fibroblast cultures in osteogenic environment. Human gingival fibroblasts (HGFs) were cultured in osteogenic differentiation medium with or without human gingival epithelial cell-derived MV stimulation. Mineralization of the cultures, localization of the MVs and mineral deposits in the HGF cultures were assessed. Gene expression changes associated with MV exposure were analyzed using gene expression profiling and real-time qPCR. Within a week of exposure, epithelial MVs stimulated robust mineralization of HGF cultures that was further enhanced by four weeks. The MVs taken up by the HGF's did not calcify themselves but induced intracellular accumulation of minerals. HGF gene expression profiling after short exposure to MVs demonstrated relative dominance of inflammation-related genes that showed increases in gene expression. In later cultures, *OSX*, *BSP* and *MMPs* were significantly upregulated by the MVs. These results suggest for the first time that epithelial cells maybe associated with the ectopic mineralization process often observed in the soft tissues.

## Introduction

Physiological mineralization is essential for correct development and function of hard tissues at proper sites, such as bone, teeth and growth plate^1^. On the other hand, mineralization also occurs in soft connective tissues, including blood vessels, skin, tendon and heart valves in pathologic conditions^2,3^. Ectopic calcification in the cardiovascular system or joints results in serious clinical outcomes because treatment options are limited^4–6^. In maxillofacial region, central ossifying fibroma, peripheral ossifying fibroma (POF) and calcifying fibrous pseudotumor have been reported as calcified fibrous lesions^7–11^. In addition, sporadic soft tissue calcifications have been found in computed tomography images when there were no clinical symptoms^12,13^. Still, the pathological mechanisms of soft tissue calcifications are not clear, although believed to be linked to environmental and clinical factors, such as aging, injury, cancer, diabetes and inflammation^2,3^. Several studies have shown a correlation between decreased bone mineral density and abnormal turnover and prevalence of ectopic soft tissue calcification^14,15^. Factors leading to pathological calcification of soft tissues however, remain elusive.

In recent years, characterization of extracellular vesicles and their biological effects have been studied in-depth for their diverse origins and release mechanisms due to their anticipated diagnostic or therapeutic value. Based on size, morphology and biological features, extracellular vesicles (EVs) are classified into exosomes, microvesicles (MVs) and apoptotic bodies^16^. While apoptotic bodies are only generated as a result of cell death, MVs and exosomes are continuously released by live cells. EVs are known to play an important role in mediating cell-to-cell communication via stabilization of labile factors secreted into the extracellular environment (i.e. peptides, proteins, lipids and microRNA)^17^, and recent studies suggest that EVs may play a role in cancer, immune regulation and human pathophysiology. EVs have also potential therapeutic value^17–21^. MVs are membrane vesicles with a diameter ranging from 100 nm to 1 µm, formed by direct outward budding of the plasma membrane. They can travel and reach distant targets in the interstitial or other body fluids while their cargo is being kept protected from degradation by the surrounding lipid membrane^22^.

We have reported previously that MVs released by keratinocytes regulate several genes in dermal fibroblasts via multiple intracellular signaling pathways, leading to increased cell migration and fibroblast-mediated angiogenesis in vitro^23^. In addition to MVs, exosomes released from oral mucosal epithelial cell sheets exhibit pro-regenerative effects on wound healing^24^. In general, EVs, especially from mesenchymal stem cells (MSCs), have been described to possess wound healing-supporting properties^25–27^. We have also reported that oral bacterial biofilms increase MV production in gingival epithelial cells (GECs)^28^. These epithelial MVs increased expression of genes associated with inflammation and matrix degradation in gingival fibroblasts^28^. Moreover, several studies have demonstrated MV-related vascular calcification. Mineralized vesicles were only abundant in the extracellular matrix (ECM) at sites of vascular calcification and not in healthy arteries. Another study reported that vascular smooth muscle cells (VSMCs) released EVs rich in osteogenic differentiation-related biomarkers under inflammatory condition^29^. EVs derived from stressed VSMCs aggregate and form microcalcification zones^30^. We have demonstrated previously that gingival fibroblasts are neural crest-derived cells that retain MSC-like properties and can differentiate to multiple lineages, including undergoing osteogenic differentiation, in vitro^31–33^. Since gingiva can develop various calcified lesions, we investigated whether MVs derived from gingival epithelial cells could regulate calcification of gingival fibroblast cultures in an osteogenic environment. Surprisingly, epithelial MVs promoted ectopic intracellular mineralization in gingival fibroblasts that was associated with early expression of inflammation related genes and late gene expression of some classical osteogenic differentiation markers and MMPs.

## Results

### Gingival epithelial cell-derived MVs induce calcification of human gingival fibroblast cultures

**Figure 1:**
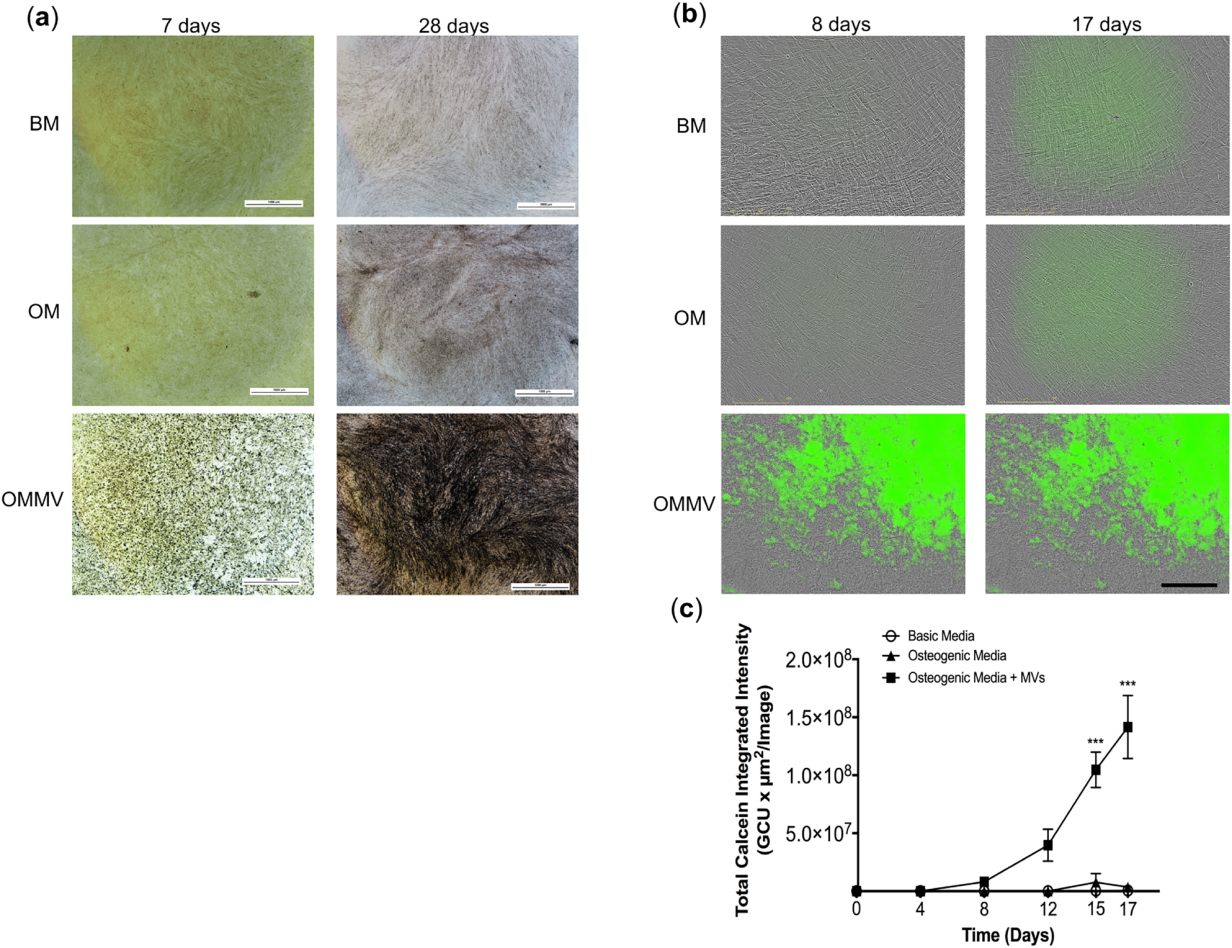
Mineralization of HGF cultures exposed to epithelial MVs. (**a**) Representative images of 7- and 28-day-old HGF cultures stained with von Kossa. Mineralized nodules were detected only in cells treated with MVs in osteogenic medium (OMMV) after one week. After a 28-day treatment, MVs promoted progressive mineralization (OMMV) compared to the control medium (OM). There was minimal mineralization in the basic medium (BM). Scale bars: 1000 µm. (**b**) IncuCyte real-time assessment of mineralization of MV-treated HGF cultures using calcein uptake for 8 and 17 days. Cells in BM and in control OM media showed only weak background fluorescence during the 17-day long experiment. In contrast, cells exposed to OMMV showed progressively increasing fluorescence from day 3 onward. Scale bars: 200 µm. (**c**) Real-time quantification of mineralization of the calcein fluorescence using the IncuCyte System. Mineralization was quantified as total calcein integrated intensity units per image (*N* = 3). ***p < 0.001.

Calcification of HGF cultures after exposure to MVs was investigated by two methods. First, the von Kossa staining was used to identify mineralization nodules after seven and twenty-eight days of exposure. Second, the mineralization of cultures was quantified in real-time using the IncuCyte system with calcein green staining. Using von Kossa staining, first clear signs of appearance of mineralized nodules were observed in 7-days-old cultures exposed to the MVs in the OM, while no nodules were identified in the untreated controls (Fig. 1a). In 28-days-old cultures, epithelial MVs further stimulated the formation of mineralized nodules (Fig. 1a). HGFs in OM also showed some mineralization but significantly less than in cultures exposed to MVs (Fig. 1a). No deposition of mineralized nodules was detected in BM at any time point.

To quantify the mineralization in the MV-exposed cultures, calcein fluorescence was used to record real-time mineral deposition over time for up to 17 days. After 7 days, calcein fluorescence started to increase in MV-treated HGF cultures in the OM medium (Fig. 1b). Calcein fluorescence then linearly increased in the treated cultures to the end of experiment at 17 days (Fig. 1b, c). OM alone induced weak mineralization while cells in the BM did not mineralize at any time point of the experiment (Fig. 1b, c).

### Analysis of the mineralized nodules

**Figure 2:**
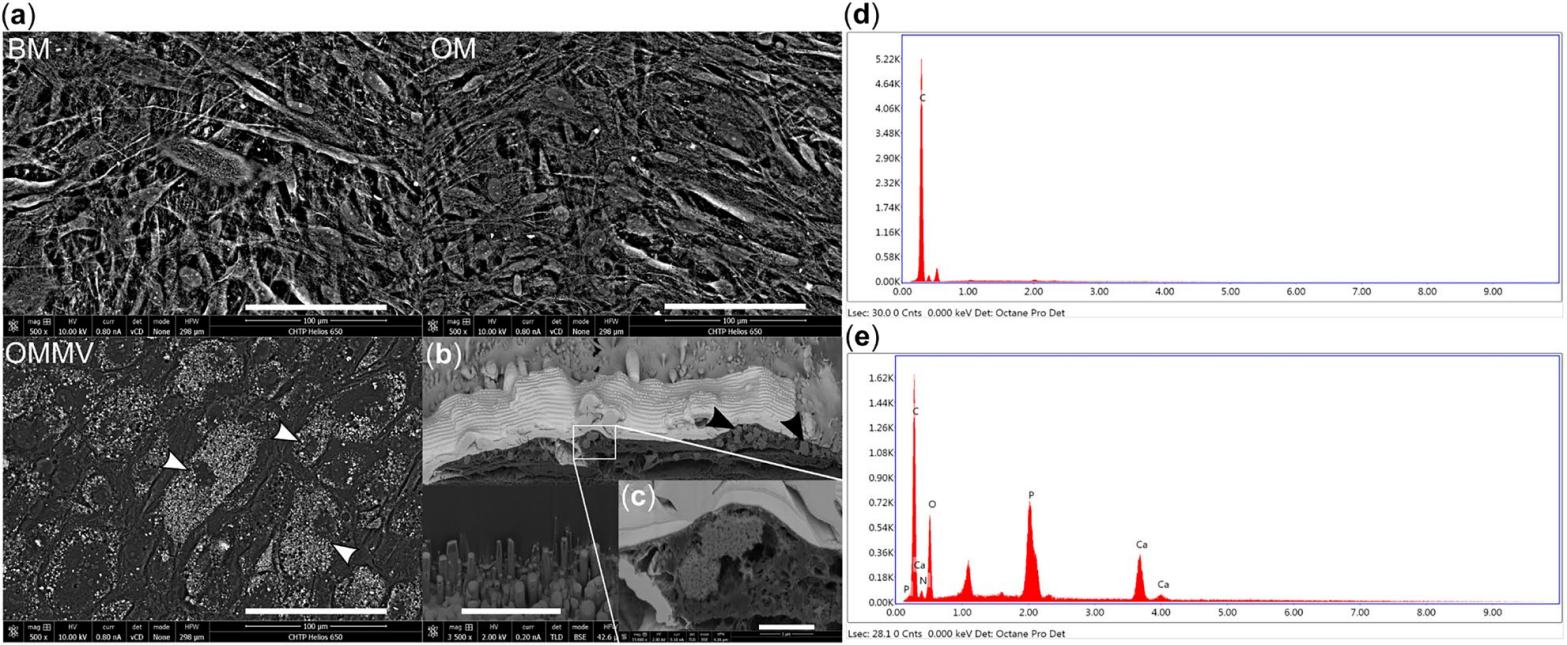
Accumulation of intracellular minerals in 28-day-old HGF cultures treated with epithelial MVs. (**a**) Representative scanning electron microscope (SEM) images of HGF cultures treated in basic medium (BM), osteogenic control medium (OM) and in OM with MVs (OMMV). Note the accumulation of electron dense material (white arrows) in the cytoplasm around the nucleus of cells in OMMV. Scale bars: 100 µm. (**b**), (**c**) focused ion beam (FIB) cross section images (cross-sectional view of cells on culture plastic) of HGF cultures in OMMV. Intracellular vesicles with high density nodules (black arrows) can be detected. Bar = 10 µm in lower magnification image and 1 µm in higher magnification image. (**d**), (**e**), The energy-dispersive X-ray spectroscopy (EDS) analysis. EDS analysis showed that both calcium and phosphorus were present in the high-density nodules in the cell interior (**e**) while control areas contained carbon only (**d**). The Ca/P ratio of these nodules was determined to be 2:1.

After we had established that epithelial MVs induce calcification of HGF cultures, we investigated whether this mineralization occurs in the ECM or intracellularly. In the SEM images, numerous nodules were detected, matching the shapes of the cells in the MV-treated cultures but not in the controls (Fig. 2a). This accumulation mimicked intracellular mineralization rather than extracellular accumulation. To confirm this observation, we used focused ion beam (FIB) to dissect the cultures in apico-coronal direction. The FIB confirmed that the nodules were located in the cytoplasm within vesicular structures (Fig. 2b, c). Using EDS, we determined that the Ca/P ratio of the mineralized nodules was 2.0 (Fig. 2e). Outside of the nodules, only carbon was detected (Fig. 2d).

### Localization of MVs in relation to mineralized nodules in the HGFs

**Figure 3:**
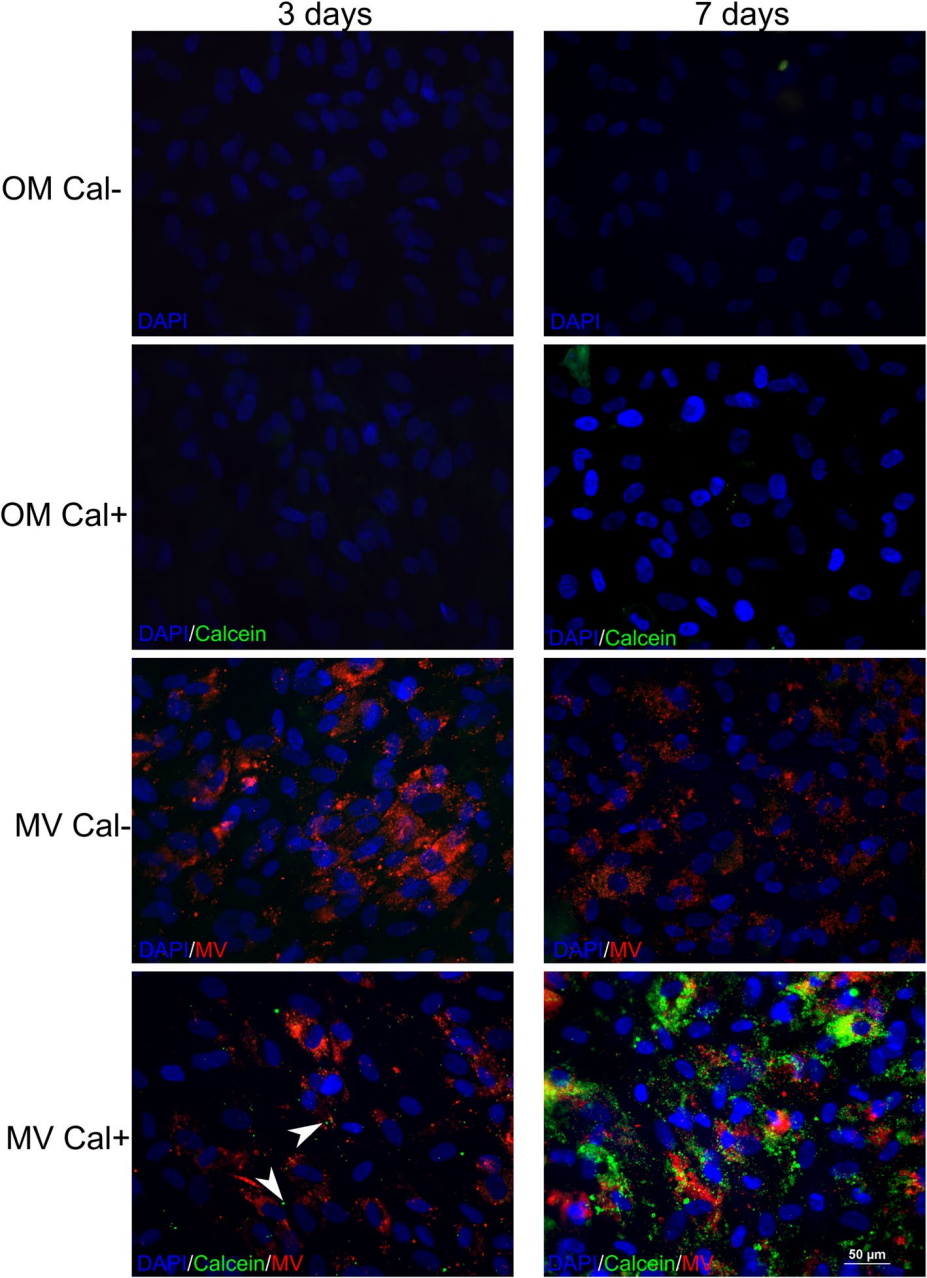
Double immunofluorescence staining of epithelial MVs and mineralized nodules in HGF cultures. Membranes of the MV were labeled with Vybrant DiI cell labeling solution (red) and minerals with calcein green. After 3-day treatment with MVs, numerous red MVs were clearly detected as dots and diffuse patches on the cell layer regardless whether calcein was present (Cal +) or not (Cal−). At this time point, only a few mineralized green dots were detected. After 7-day treatment with MVs, the number of mineral nodules significantly increased around nuclei. However, no co-location of the MVs and the mineralized nodules were detected. HGFs without MVs showed no green calcein positive nodules. Scale bars: 50 µm.

To find out whether the mineralization is directly initiated by the MV intake, we performed double staining of the MVs and nodules at the early stages of mineralization. After three days of exposure to the red fluorescent MVs, the MVs were clearly detected as dots and diffuse patches on the cell layer (Fig. 3). At this time point, only a few calcein-fluorescent green dots were detected scattered at the periphery of the cells and not co-localized with MVs (Fig. 3). After a 7-day treatment with MVs, the number of calcium nodules significantly increased around nuclei (Fig. 3). However, no co-location of the MVs and the mineralized nodules were detected (Fig. 3). HGFs without MVs showed no green calcein positive nodules at this time point.

### Gene expression profiling

**Figure 4:**
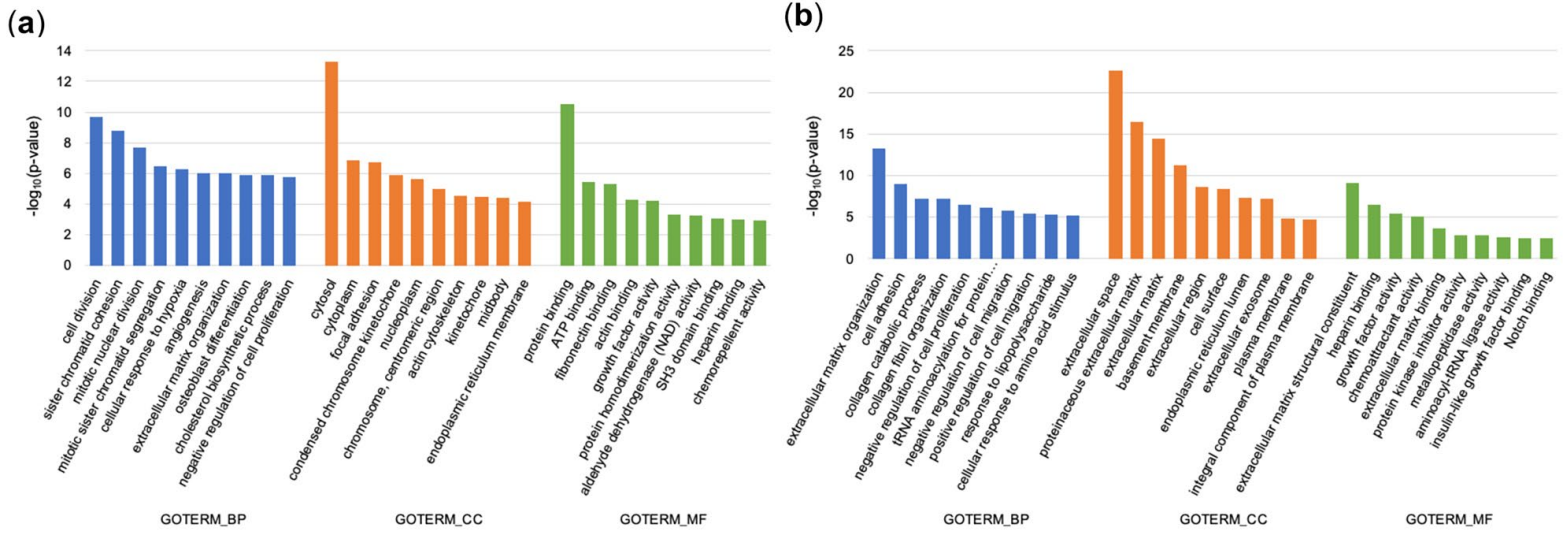
Profiling of HGF genes differentially expressed after 1- and 3-day treatment with epithelial MVs in osteogenic medium. (**a**), (**b**) Gene ontology enriched annotations of biological process (GOTERM_BP), cellular component (GOTERM_CC) and molecular function (GOTREM_MF) associated with differentially expressed genes (DEGs) on HGFs in response to MV stimulation after 1 day (**a**) and 3 days (**b**) using The Database for Annotation, Visualization, and Integrated Discovery (DAVID). After 1-day stimulation, annotation about cell cycle, cell component and protein binding were enriched. On the other hand, genes related extracellular matrix (ECM) component were enriched after 3-days stimulation. Three biological replicates were included in the analysis.

**Figure 5:**
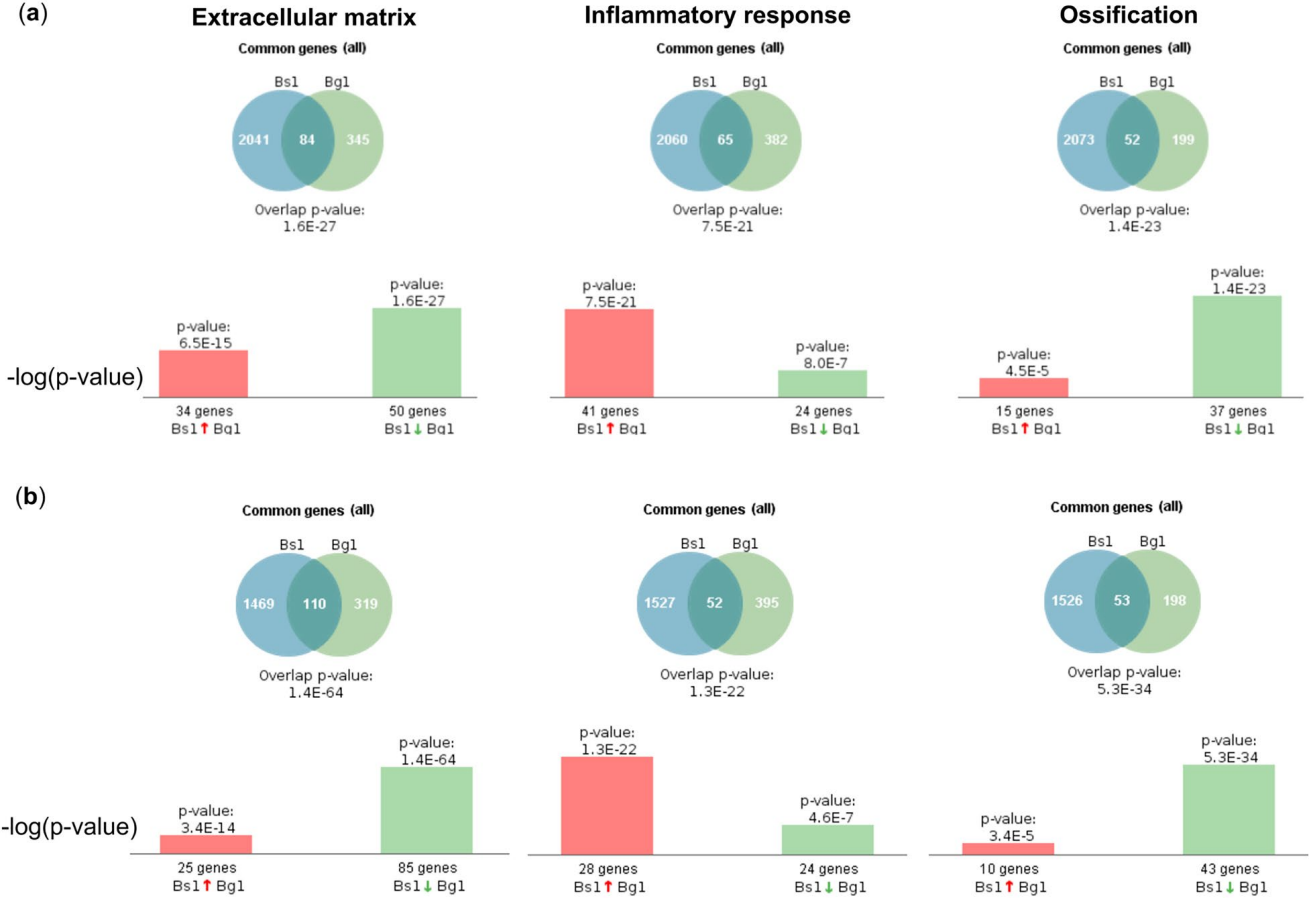
Profiling of HGF genes related to extracellular matrix (ECM), Inflammatory response (INF) and Ossification (OSS) differentially expressed after 1- and 3-day treatment with epithelial MVs in osteogenic medium. (**a**), (**b**) differentially expressed genes (DEGs) were analyzed in groups related to ECM, INF and OSS after (**a**) 1 day and (**b**) 3 days stimulation using BaseSpace Correlation Engine. Genes related Inflammatory response were increased after 1- and 3-day stimulation. Genes related ECM were downregulated on 1 and 3 days. Ossification-related genes were mainly down-regulated. Three biological replicates were included in the analysis.

To investigate changes associated with the mineralization process induced by the epithelial MVs, we performed gene profiling of the early changes in 1- and 3-day-old cultures and analyzed the expression of osteogenic differentiation associated genes in cultures showing MV-induced mineralization on days 7 and 28. Total of 2125 and 1579 genes were differentially expressed in HGFs with fold-change ≥ 1.5 and at p < 0.05 in response to MV stimulation on days 1 and 3, respectively. The top 10 enriched gene ontology annotations of biological process (GOTERM_BP), cellular component (GOTERM_CC) and molecular function (GOTREM_MF) associated with differentially expressed genes (DEGs) are shown in Fig. 4a and b. After 1-day stimulation with the MVs, the categories related to cell proliferation, cytoplasmic components and protein binding were significantly regulated in HGFs (Fig. 4a). After 3-days of stimulation, the categories in treated cells had shifted to ECM components and organization (Fig. 4b). DEGs were further analyzed in groups related to ECM, Inflammatory response (INF) and Ossification (OSS) (Fig. 5a and b). In the ECM group, 84 and 110 genes were differentially regulated by MVs after one- and three-days stimulation, respectively (50 genes down and 34 up for one day and 85 genes down and 25 up for three days). In the INF group, 65 and 52 genes were regulated by the MVs with upwards trend in the majority of the genes (*N* = 41 and 28). In the OSS group, 52 and 53 genes were significantly regulated by the MVs with majority showing downregulation (37 and 43 genes) (Fig. 5a and b). When individual up-regulated genes were analyzed in the ECM, INF and OSS groups, it clearly showed the relative dominance of INF related genes (such as *IL-8*, *CXCL1*, *C3*, *IL-6*, *SAA1*, *SAA2* and *PTX3*) that showed strong increases in gene expression (Tables 1 and 2). Not surprisingly, the TNF and NF-κB signaling pathways were concurrently increased (Table 3).

**Table 1:** Top 10 upregulated genes of differentially expressed genes (DEGs) on HGFs with epithelial MV stimulation related to (a) Extracellular matrix (ECM), (b) Inflammatory response (INF), and (c) Ossification (OSS) after 1-day culture. In ECM group, *MMP1* and *MMP3* were upregulated with MV stimulation. Genes related inflammation (*IL-8*, *CXCL1* and *C3*) were highly upregulated. Also, *SAA1* and *SAA2* were upregulated. Among OSS, *TNC*, *FOXC1* and *MSX2* were upregulated.

Gene	EntrezGene ID	Description	Fold change
(**a**)			
*FLRT2*	23,768	fibronectin leucine rich transmembrane protein 2	3.579
*TIMP3*	7078	TIMP metallopeptidase inhibitor 3	3.138
*MMP1*	4312	matrix metallopeptidase 1 (interstitial collagenase)	3.103
*TFPI2*	7980	tissue factor pathway inhibitor 2	2.959
*ELN*	2006	elastin	2.863
*VEGFA*	7422	vascular endothelial growth factor A	2.841
*ABI3BP*	25,890	ABI family, member 3 (NESH) binding protein	2.671
*NTN1*	9423	netrin 1	2.367
*PXDN*	7837	peroxidasin homolog (Drosophila)	2.244
*MMP3*	4314	matrix metallopeptidase 3 (stromelysin 1, progelatinase)	2.205
(**b**)			
*IL8*	3576	interleukin 8	2379.759
*CXCL1*	2919	chemokine (C-X-C motif) ligand 1 (melanoma growth stimulating activity, alpha)	645.543
*C3*	718	complement component 3	41.585
*PTGS2*	5743	prostaglandin-endoperoxide synthase 2 (prostaglandin G/H synthase and cyclooxygenase)	34.940
*SAA2*	6289	serum amyloid A2	32.067
*TNFAIP6*	7130	tumor necrosis factor, alpha-induced protein 6	30.898
*IL6*	3569	interleukin 6 (interferon, beta 2)	22.328
*SAA1*	6288	serum amyloid A1	12.181
*NFKBIZ*	64,332	nuclear factor of kappa light polypeptide gene enhancer in B-cells inhibitor, zeta	11.631
*PTX3*	5806	pentraxin 3, long	9.052
(**c**)			
*STC1*	6781	stanniocalcin 1	2.998
*IARS*	3376	isoleucyl-tRNA synthetase	2.398
*TNC*	3371	tenascin C	2.100
*IGFBP3*	3486	insulin-like growth factor binding protein 3	1.980
*EPHA2*	1969	EPH receptor A2	1.883
*NAB1*	4664	NGFI-A binding protein 1 (EGR1 binding protein 1)	1.856
*FOXC1*	2296	forkhead box C1	1.851
*MSX2*	4488	msh homeobox 2	1.771
*MMP14*	4323	matrix metallopeptidase 14 (membrane-inserted)	1.770
*COL13A1*	1305	collagen, type XIII, alpha 1	1.760

**Table 2:** Top 10 upregulated genes of differentially expressed genes (DEGs) on HGFs MV stimulation related to (a) Extracellular matrix (ECM), (b) Inflammatory response (INF), and (c) Ossification (OSS) after 3-day culture. In the ECM group, *MMP1* and *MMP3* were upregulated with MV stimulation. Genes related INF (*IL8*, *CXCL1*, *C3*, *SAA2*, *SAA1* and *PTX3*) continued to be highly upregulated.

Gene	EntrezGene ID	Description	Fold Change
(**a**)			
*TFPI2*	7980	tissue factor pathway inhibitor 2	8.921
*TIMP3*	7078	TIMP metallopeptidase inhibitor 3	6.786
*MMP1*	4312	matrix metallopeptidase 1 (interstitial collagenase)	4.149
*MMP3*	4314	matrix metallopeptidase 3 (stromelysin 1, progelatinase)	2.925
*NOV*	4856	nephroblastoma overexpressed	2.854
*ABI3BP*	25,890	ABI family, member 3 (NESH) binding protein	2.599
*VEGFA*	7422	vascular endothelial growth factor A	2.517
*PXDN*	7837	peroxidasin homolog (Drosophila)	2.387
*MMP10*	4319	matrix metallopeptidase 10 (stromelysin 2)	2.300
*ADAMTS6*	11,174	ADAM metallopeptidase with thrombospondin type 1 motif, 6	2.277
(**b**)			
*IL8*	3576	interleukin 8	386.981
*CXCL1*	2919	chemokine (C-X-C motif) ligand 1 (melanoma growth stimulating activity, alpha)	184.991
*C3*	718	complement component 3	38.513
*IL6*	3569	interleukin 6 (interferon, beta 2)	12.166
*CCL2*	6347	chemokine (C–C motif) ligand 2	11.648
*CCL11*	6356	chemokine (C–C motif) ligand 11	11.062
*PTX3*	5806	pentraxin 3, long	6.576
*BDKRB2*	624	bradykinin receptor B2	6.261
*PTGS2*	5743	prostaglandin-endoperoxide synthase 2 (prostaglandin G/H synthase and cyclooxygenase)	5.796
*SAA2*	6289	serum amyloid A2	5.380
*TNFAIP6*	7130	tumor necrosis factor, alpha-induced protein 6	4.836
(**c**)			
*IARS*	3376	isoleucyl-tRNA synthetase	3.214
*DDX21*	9188	DEAD (Asp-Glu-Ala-Asp) box helicase 21	2.087
*KLF10*	7071	Kruppel-like factor 10	1.849
*NAB1*	4664	NGFI-A binding protein 1 (EGR1 binding protein 1)	1.685
*FSTL3*	10,272	follistatin-like 3 (secreted glycoprotein)	1.683
*GPNMB*	10,457	glycoprotein (transmembrane) nmb	1.642
*FOXC1*	2296	forkhead box C1	1.635
*MYBBP1A*	10,514	MYB binding protein (P160) 1a	1.634
*TNC*	3371	tenascin C	1.616
*RSL1D1*	26,156	ribosomal L1 domain containing 1	1.565

**Table 3:** Top 10 of Kyoto Encyclopedia of Genes and Genomes (KEGG) pathway analysis of differentially expressed genes (DEGs) in MV stimulated HGFs compared to control after 1 day (a) and 3 days (b) using The Database for Annotation, Visualization, and Integrated Discovery (DAVID). At 1 day, pathways of cell cycle were highly upgraded. TNF signaling pathway was up regulated both after 1-day and 3-days stimulation. NF-κB signaling pathway was upregulated after 3 days of culture.

1 days up	count	*P*-value
(**a**)		
Cell cycle	32	3.68E-10
p53 signaling pathway	17	1.36E-05
TNF signaling pathway	20	1.77E-04
Oocyte meiosis	20	2.90E-04
Aminoacyl-tRNA biosynthesis	14	6.67E-04
Small cell lung cancer	16	8.93E-04
Fanconi anemia pathway	12	1.09E-03
FoxO signaling pathway	20	2.99E-03
Transcriptional misregulation in cancer	23	3.72E-03
HTLV-I infection	31	4.22E-03
Toxoplasmosis	17	4.82E-03

3 days up	count	*P*-value
(**b**)		
Aminoacyl-tRNA biosynthesis	15	5.63E-07
Biosynthesis of amino acids	13	4.87E-05
Small cell lung cancer	14	6.03E-05
One carbon pool by folate	7	1.40E-04
TNF signaling pathway	14	6.32E-04
Biosynthesis of antibiotics	21	7.45E-04
PI3K-Akt signaling pathway	28	1.76E-03
NF-kappa B signaling pathway	11	3.96E-03
Epstein-Barr virus infectio	13	6.04E-03
HIF-1 signaling pathway	11	7.96E-03

**Figure 6:**
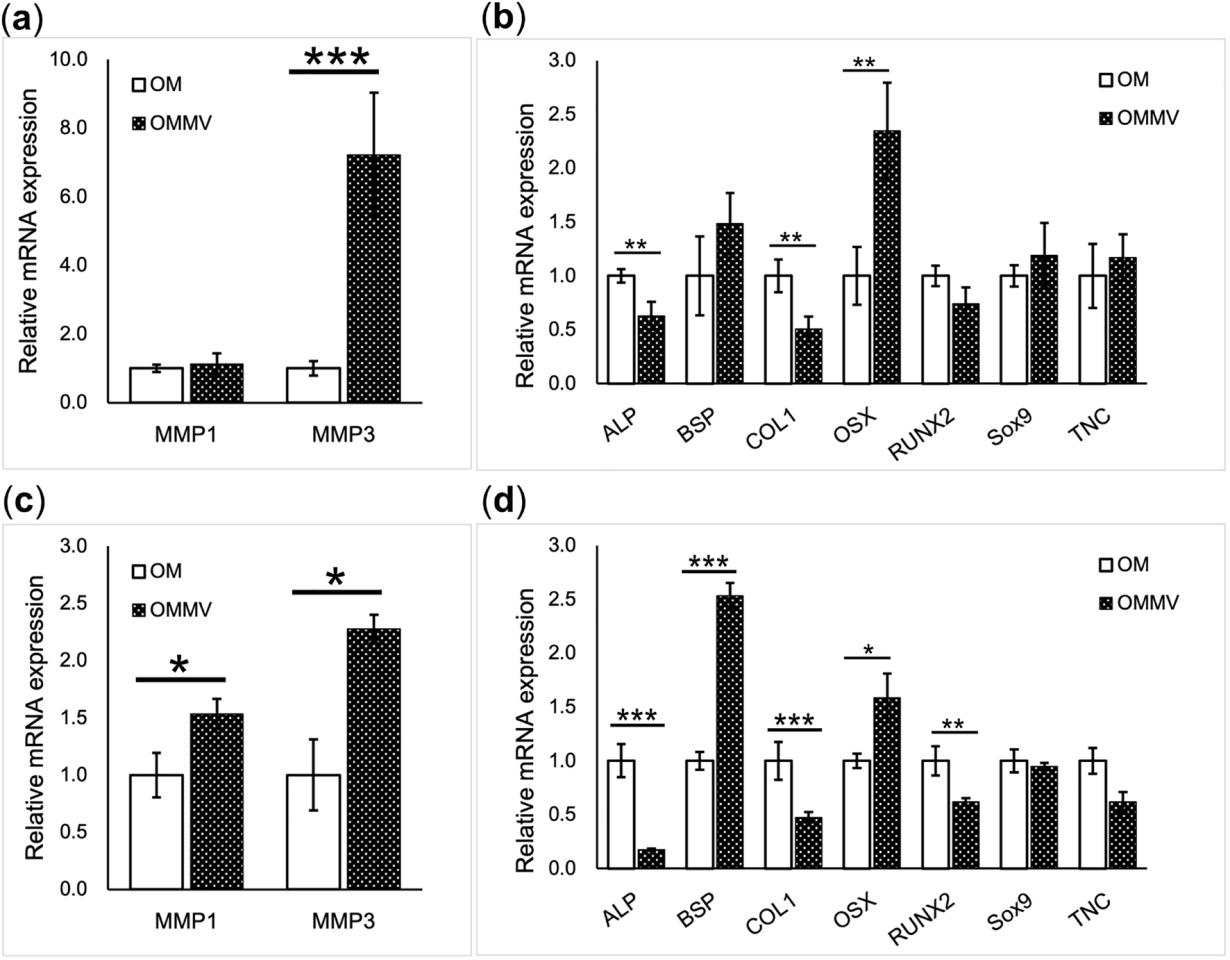
The effect of epithelial MVs on HGFs gene expression after 7- and 28-day stimulation. Gene expressions were analyzed by RT-qPCR. Cells without MVs (OM) were used as control. Results show mean ± standard error of the mean (SEM) from triplicate experiments (*p < 0.05, **p < 0.01, ***p < 0.001). The genes are grouped by function; (**a**), (**c**) *MMPs*, and (**b**), (**d**) osteogenic differentiation-related genes after 7-days (**a**), (**b**) and 28-day (**c**), (**d**) stimulation. Expression of *MMP-3 and OSX* was highly up-regulated (**a**) while expression of *ALP* and *COL I* was downregulated after 7-days stimulation (**b**). After 28-day culture, *MMP1* and *MMP3* were significantly up-regulated with MV stimulation (**c**). Among osteogenic differentiation-related genes, *OSX* and *BSP* were up-regulated with MV stimulation. On the other hand, expression of *ALP*, *RUNX2* and *COL I* was downregulated (**d**).

Long-term changes in expression of selected HGF genes by epithelial MVs were investigated by RT-qPCR. The results at day 7 showed that the relative expression *MMP-3* was significantly upregulated while the expression of *MMP-1* was not changed (Fig. 6a). Among osteogenic differentiation-related genes, *OSX* was significantly upregulated by the MVs. However, the expression of *ALP* and *COL1* were significantly downregulated, and there was no change in *RUNX-2* and *SOX9* expression (Fig. 6b). After 28 days in the OM, the relative expression of *MMP1* and *MMP3* was significantly upregulated by epithelial MVs (Fig. 6c). Among osteogenic differentiation-related genes, expression of *OSX* and *BSP* continued to be upregulated (Fig. 6d). However, expression of *ALP*, *RUNX-2* and *COL1* was downregulated by the MVs (Fig. 6d).

## Discussion

Pathologic ectopic calcification of soft tissues remains a significant clinical problem. Calcifications occur in blood vessel walls, in many connective tissues and in benign and malignant tumors. The molecular mechanisms of soft tissue calcification are relatively poorly understood. We report in this paper that epithelia-driven mechanisms, including MVs, could be involved in a potential pathway leading to calcification of soft tissues. The key observations of our study show that epithelial MVs strongly induced mineralization of HGF cultures, and this was associated with remarkable early upregulation of genes linked to inflammation and MMP activity. Furthermore, some of the genes associated with osteogenic differentiation such as *OSX*^34^ showed up-regulation with advanced mineralization that was largely contained in vesicles in the cytoplasm. Osteogenic differentiation is driven by key transcription factors *RUNX-2* and *OSX* that shift the profile of gene expression to support matrix deposition (type I collagen expression) and mineralization mediated by *ALP*^35^. HGFs treated with GEC-MVs in osteogenic conditions showed consistent up-regulation of *OSX*. However, *RUNX-2* was not altered at early time point and reduced in later time point which is curious given that *OSX* acts downstream of *RUNX-2*^36^. In addition, the expression of *ALP* and type I collagen were both strongly downregulated by the MVs. Therefore, intracellular mineralization does not seem to follow gene expression profile associated with typical osteogenic differentiation. ALP is present in the MVs (data in file for Bi et al., 2016^28^) and could be provided for the intracellular mineralization process.

EVs, including exosomes and MVs, are highly heterogenous group of vesicles produced by practically all cell types^37^. Before studies on EVs were common, vesicles associated with endochondral bone formation were identified and called matrix vesicles^38,39^. The size of these vesicles range between 50 and 400 nm and could, therefore, represent a mixture of exosomes and MVs^40,41^. MVs produced by osteogenic cells can accumulate Ca^2+^ and P intracellularly, and these vesicles serve as sites of mineral nucleation^42^. Epithelial MVs were internalized by the HGFs but did not seem to directly mineralize. We also did not observe any mineralization in HGF cultures that were exposed to epithelial MVs in the basic medium supporting the view that these MVs stimulate mineralization through an indirect process that is likely associated with their cargo and a suitable environment. We have previously analyzed the protein content of MVs derived from the same gingival epithelial cell line used in the present study^28^. There are over 2000 proteins present in the epithelial MVs, and it is likely that, in osteogenic environment, some could be recycled to form new vesicles that have Ca^2+^-binding capacity. For example, epithelial MVs contain several annexins that can bind both Ca^2+^ and phospholipids and can potentially act as nucleation sites for mineralization when released into the target cells. A previous study has demonstrated that EVs derived from mineralizing osteoblasts contain more annexins than from non-mineralizing osteoblasts^42^. The reasons why the newly formed vesicular structures that were mineralized with material having a Ca^2+/^P ratio close to hydroxyapatite were not released from the cells remains to be further investigated. Biogenesis and release of EVs is a complex process and involves often multiple steps and sorting machineries^37^ that may be deficient in HGFs, leading to trapping of the vesicles in the intracellular milieu. Mitochondrial participation in mineralization has been proposed in several studies^43–45^. We have not confirmed mitochondrial role for epithelial-driven and MV-associated intracellular mineralization of HGFs, and it warrants further studies. However, intracellular accumulation of mineral-containing vesicles has been observed in several other studies using different cell lines^46–48^.

Epithelial MVs caused a strong and early response of the expression of genes related to inflammation and matrix metalloproteinase activity. These findings support our previous findings that showed MV-induced gene expression of IL-6, IL-8, MMP-1 and MMP-3 in HGFs^28^. MVs from epidermal keratinocytes also stimulate the same genes in dermal fibroblasts, supporting the notion that this signaling machinery stands as universal communication mechanism between epithelial cells and fibroblasts^23^. Pro-inflammatory signals are likely to initiate normal bone healing and promote osteogenesis, but chronic inflammation is detrimental for bone formation^49–52^. Inflammation is also linked to extraosseous mineralization in pathological conditions, such as atherosclerosis, bone metastasis and others^53,54^. Serum amyloid A proteins 1 and 2 (SAA1/2) are released by liver in response to inflammation but also produced locally to regulate inflammatory cytokine release through activation of toll-like receptors TLR2 and TLR4^55,56^. In mesenchymal stem cells, both autocrine and paracrine SAA1/2 stimulate inflammatory cytokine expression and enhanced mineralization^57^. In addition, the expression of inflammatory cytokines, such as IL-6 and IL-8, is highly elevated during osteogenic differentiation of bone marrow mesenchymal stem cells^58^. Interestingly, expression of both SAA1 and 2 were significantly increased in HGFs by the MVs and could, therefore, mediate strong up-regulation of cytokines such as Il-6, IL-8 and CXCL1. Another inflammation-related gene that was upregulated by MVs was *PTX3* (long pentraxin 3). Intriguingly, PTX3 appears to play a role in regulation of both normal and ectopic mineralization by promoting cell differentiation and mineral crystal formation^59^. Particularly, calcifications in normal and cancerous tissue in the breast, in prostate cancer and in atheromas are associated with high PTX3 expression^60–62^. As expected, TNF and NF-B signaling pathways were increased in MV-treated HGFs. Interestingly, a low dose of TNF stimulation has been reported to induce persistent Wnt expression through NF-κB and JNK signaling pathways, resulting in bone formation^63^. Our previous studies have shown that JNK signaling pathway is activated by epithelial MVs in HGFs and dermal fibroblasts^23,28^. Therefore, NF-κB and JNK signaling could participate in MV-induced mineralization of HGF cultures.

In the present study, *MMP1* and *MMP3* were strongly upregulated by epithelial MVs, corroborating our previous observations with dermal and gingival fibroblasts^23,28^. Epithelial MVs regulate MMPs mainly through ERK1/2 signaling. Increased expression of MMPs, including MMP-1 has been also found in bone marrow MSCs during osteogenic differentiation^64^. Other MMPs, such as MMP-2, MMP-9 and MT1-MMP, are also stimulated during early mineralization^64,65^. MMP-1 may directly promote osteogenic differentiation through ERK and JNK pathways^66^. MMP-1 stimulation of bone marrow MSCs upregulated osteogenic differentiation markers (*RUNX2, OSX, OPN* and *OCN*) and promoted mineralization^66^. Whether other MMPs could also directly stimulate osteogenic differentiation remains unknown. Overall, a number of MMPs play a crucial role in bone development and pathology but their role is multifaceted^67^.

Possible limitations of the present study include the use of MVs from spontaneously immortalized human gingival epithelial cells. It is well known that the cargo in the EVs can vary depending on cell types and pathological conditions^46^. However, epithelial MVs from spontaneously immortalized gingival and epidermal keratinocytes as well as from primary keratinocytes similarly induce MMP and cytokine expression on fibroblasts from different origins^23,28^, suggesting that at least some of the cargo proteins are shared. In addition, we were not able to determine the molecules involved in the induction of the mineralization and why the minerals were retained in the cytoplasm. These interesting questions will need to be answered in a separate future study.

In conclusion, the present study shows that gingival epithelial-derived MVs induce rapid mineralization of gingival fibroblast cultures in osteogenic conditions. This process is associated with early upregulation of inflammation and MMP activity-related genes that could positively regulate the osteogenic differentiation. Remarkably, that the mineralization remained largely intracellular, warrants further investigation.

## Methods

### Cell culture

Spontaneously immortalized human gingival epithelial cell (GEC) line was established in the authors’ laboratory^68^. This cell line has partial triploid phenotype but no known mutations. Normal primary human gingival fibroblast strain (GFBL-HN; referred here as HGFs) was isolated from healthy attached gingiva of a healthy 18-year-old female^69^. This cell strain possesses an average phenotype of several primary gingival fibroblast strains tested in our previous study^69^. Both cell types were maintained in basic medium (BM) containing Dulbecco’s modified Eagle’s medium (Gibco, Life Technologies, Grand Island, NY, USA) supplemented with 23 mM sodium bicarbonate, 20 mM HEPES, 1% antibiotics (50 μg/mL of streptomycin sulfate, 100 U/mL of penicillin; Gibco), and 10% heat-inactivated fetal bovine serum (FBS; Gibco).

### MV collection

MV collection was performed as previously described^28^. Briefly, confluent GECs were rinsed with phosphate-buffered saline (PBS) and cultured in FBS-free medium for 48 h. Conditioned medium from the cell cultures was collected and centrifuged (Sorvall RC 5B Plus, Mandel Scientific; manufactured by DuPont, Newtown, CT, USA) at 4℃, first at 3,000 g for 15 min to remove cellular debris and then at 25,000 g for 30 min to collect MVs. MV pellets were rinsed once with PBS, homogeneously re-suspended in FBS-free medium and kept at 4℃ until used. Total MV protein content was measured with Bio-Rad Protein Assay reagent (Bio-Rad Laboratories, Hercules, CA, USA) and spectrophotometry at 570 nm and used for standardizing the vesicle amounts in the experiments. Characterization of MVs (physical size, proteomics analysis) produced by the GEC line used has been published in a previous study^28^.

### HGF stimulation by epithelial MVs in osteogenic conditions

HGFs were seeded in 24-well plates (2 × 10^5^ cells per well) for 48 h. Then, the medium was changed to osteogenic differentiation medium [OM; BM supplemented with 50 µg/ml of ascorbic acid (Sigma-Aldrich, St Louis, MI, USA), 100 nM dexamethasone (Sigma- Aldrich), 100 nM vitamin D3 (Sigma-Aldrich), and 10 mM ß-glycerophosphate (Sigma- Aldrich)] and MVs were added to the final concentration of 30 μg protein/ml. Medium was changed and fresh MVs were added every 72 h.

### Staining of mineralized nodules by von Kossa

Von Kossa staining was performed as previously reported^70^. In brief, the cells were fixed in 4% formaldehyde (Fisher Scientific, Fair Lawn, NJ, USA) after 7- and 28-days culture periods and incubated with 2% silver nitrate (Fisher Scientific) in dark for 10 min and then exposed to bright light for 15 min. The plates were then washed with distilled water and dehydrated in 100% ethanol. The samples were then examined by light microscopy^71^.

### Calcification of cultures measured by IncuCyte real-time imaging

For real-time imaging of calcification, 10 mM calcein green (Sigma-Aldrich) solution, which fluoresces when bound to calcium crystals, was prepared in 0.1 M NaOH, and the solution was further diluted to 1 mM and sterile-filtered. Calcein solution was added directly to the differentiation medium in final concentration of 1 μM^72^. The culture medium was changed every three days. The green fluorescence mask of GCU 270 was set in order to exclude background noise to avoid artifacts^72^. The images were taken in real-time, using an automated Incucyte^tm^ S3 live-cell imaging system (Sartorius Corporation, Edgewood, NY, USA) at a series of time points up to 17 days.

### Sample preparation for SEM

HGFs were cultured on 10 mm tissue culture plastic coverslips (Sarstedt, Newton, NC, USA) in 24-well plates for four weeks, fixed with 2.5% glutaraldehyde (EM grade, Electron Microscopy Sciences (EMS), Hatfield, PA, USA), postfixed with 1% osmium tetroxide (EMS) and dehydrated in a graded series of ethanol followed by critical point drying (Samdri-795; Tousimis Research Corporation, Rockville, MD, USA). The coverslips were mounted on stubs and coated with 20-nm carbon (Med020, Leica Microsystems Inc, Concord, ON, Canada). Cells were visualized by SEM (Helios 650 Nanolab dual beam, FEI, Hillsboro, OR, USA) using the backscattered electrons (BSE) imaging mode (atomic number contrast) and electron energy sectioning^73^. Once a suitable area was identified, a cross section was created with the Focused Ion Beam (FIB), exposing the interior of the cells. The BSE imaging mode was used to distinguish the cell and intracellular calcified vesicles based on atomic number contrast. To confirm the presence of Ca (L alpha of 3.9 keV) and P (K alpha 2.013) areas of high density were analyzed using EDX point analysis (Octane Pro Detector, EDAX, Mahwah, NJ, USA).

### Double staining of MVs and mineral deposits

To analyze direct relationship between MVs and calcification, the GECs were labeled using Vybrant DiI cell labeling solution (1:200; Invitrogen, Eugene, OR, USA) for 1 h. Labeled MVs were collected as above. HGFs were seeded on gelatin (0.2%)-coated glass coverslips in BM. After 48 h, the medium was changed to OM supplemented with 1 μM calcein green, and Dil-labeled MVs were added. MVs were added only once at day 0. Medium was changed at day 4. At each time point, the cells were fixed with 4% formaldehyde at room temperature for 20 min and the nuclei then stained with 300 nM DAPI (Molecular Probes Inc., Eugene, OR, USA) in PBS for 15 min. Samples were mounted with Immuno-mount solution (Thermo Scientific, Pittsburgh, PA, USA) and examined using an Axioplan II Fluorescent microscope (Carl Zeiss Inc., Jena, Germany). The images were captured using Northern Eclipse software (Empix Imaging, Mississauga, ON, Canada).

### Gene expression profiling

HGFs were seeded in 6-well plates (2 × 10^5^ cells per well) in BM for 48 h, after which the medium was changed to OM and MVs were added as above. After a 1- or 3-day culture, total RNA was extracted using Purelink RNA Mini kit (Thermo Scientific) according to the manufacturer’s protocol and assessed for purity by the RNA/DNA Calculator (GeneQuant Pro; Amersham Biosciences, Little Chalfont, Buckinghamshire, UK). The quality of RNA samples was assessed using Agilent Bioanalyzer (Agilent Technologies, Santa Clara, CA, USA), and samples with RNA Integrity Number (RIN) ≥8 were used for further analysis^74^. Qualifying samples were then prepped following the standard protocol for the NEBnext Ultra ii Stranded mRNA (New England Biolabs, Ipswich, MA, USA). Sequencing was performed on the Illumina NextSeq 500 (Illumina Inc. San Diego, CA, USA) with Paired End 42 bp × 42 bp reads. De-multiplexed read sequences were then aligned to the reference sequence using STAR aligners (https://www.ncbi.nlm.nih.gov/pubmed/23104886). Assembly and differential expression were estimated using Cufflinks (http://cole-trapnell-lab.github.io/cufflinks/) through bioinformatics apps available on Illumina Sequence Hub. Gene abundances were normalized by calculating Fragments Per Kilobase of Exon Per Million Fragments Mapped (FPKM). Differentially expressed genes (DEGs) were identified with a Benjamini–Hochberg adjusted q-value of less than 5% and absolute fold-change of ≥ 1.5. The data were analyzed by the BaseSpace Correlation Engine (https://basespace.illumina.com, Illumina, Cupertino, CA) and The Database for Annotation, Visualization, and Integrated Discovery (DAVID) Resources 6.7 (at http://david.abcc.ncifcrf.gov/) using default setting. Gene ontology (GO) and Kyoto Encyclopedia of Genes and Genomes (KEGG) pathway analysis was used to evaluate the function of DEGs in two groups.

### Quantitative reverse transcription PCR (RT-qPCR)

RT-qPCR was performed as previously described^75^. Briefly, total RNA was obtained as above. Total RNA (1 μg) was reverse-transcribed with high-capacity cDNA reverse transcription kit (Applied Biosystems, Life Technologies, Grand Island, NY, USA), according to the manufacturer’s instructions, and Mastercycler Gradient 5331 Reverse-Transcriptase PCR Instrument (Eppendorf AG, Hamburg, Germany). The cDNA was diluted to a concentration at which the threshold-cycle value was well within the range of its standard curve. cDNA (5 μl) was mixed with 10 μl of 2 × iQ SYBR Green I Supermix (Bio-Rad) and 5 pmol of primers in 96-well plate wells. Asparagine-linked glycosylation 9 (*ALG9*) and glyceraldehyde- 3-phosphate dehydrogenase (*GAPDH*) were used as reference genes. Real-time qPCR amplification was performed using the CFX96 system (Bio-Rad Laboratories). The data were analyzed and are presented according to the comparative Ct method (CFX Manager Software, version 2.1; Bio-Rad Laboratories). PCR primers used are listed in the supplementary Table S1.

### Statistical analysis

All data are expressed as mean ± standard error of the mean (SEM) from at least three independent experiments. Statistical analysis was performed using SPSS 24.0 software. Statistical differences were evaluated by Student’s t-test for paired comparisons or by one-way ANOVA followed by Tukey’s post hoc test for multiple comparisons. Statistical analysis for RT-qPCR data was done using log2-transformed data. P values < 0.05 were considered as statistically significant.

## Supplementary Information

Supplementary Information.
